# Regulation of caspase-3 processing by cIAP2 controls the switch between pro-inflammatory activation and cell death in microglia

**DOI:** 10.1038/cddis.2014.514

**Published:** 2014-12-11

**Authors:** E Kavanagh, J Rodhe, M A Burguillos, J L Venero, B Joseph

**Affiliations:** 1 Department of Oncology-Pathology, Cancer Centrum Karolinska, R8:03, Karolinska Institutet, Stockholm, Sweden; 2 Departamento de Bioquímica y Biología Molecular, Facultad de Farmacia, Universidad de Sevilla and Instituto de Biomedicina de Sevilla (IBiS), Sevilla, Spain

## Abstract

The activation of microglia, resident immune cells of the central nervous system, and inflammation-mediated neurotoxicity are typical features of neurodegenerative diseases, for example, Alzheimer's and Parkinson's diseases. An unexpected role of caspase-3, commonly known to have executioner role for apoptosis, was uncovered in the microglia activation process. A central question emerging from this finding is what prevents caspase-3 during the microglia activation from killing those cells? Caspase-3 activation occurs as a two-step process, where the zymogen is first cleaved by upstream caspases, such as caspase-8, to form intermediate, yet still active, p19/p12 complex; thereafter, autocatalytic processing generates the fully mature p17/p12 form of the enzyme. Here, we show that the induction of cellular inhibitor of apoptosis protein 2 (cIAP2) expression upon microglia activation prevents the conversion of caspase-3 p19 subunit to p17 subunit and is responsible for restraining caspase-3 in terms of activity and subcellular localization. We demonstrate that counteracting the repressive effect of cIAP2 on caspase-3 activation, using small interfering RNA targeting cIAP2 or a SMAC mimetic such as the BV6 compound, reduced the pro-inflammatory activation of microglia cells and promoted their death. We propose that the different caspase-3 functions in microglia, and potentially other cell types, reside in the active caspase-3 complexes formed. These results also could indicate cIAP2 as a possible therapeutic target to modulate microglia pro-inflammatory activation and associated neurotoxicity observed in neurodegenerative disorders.

## Introduction

Microglia cells are the resident immune cells of the central nervous system, constantly screening the brain environment. They express surface receptors to detect changes in their environment due to brain damage or infections. An important family of these sensors is the toll-like receptor (TLR) family.^1^ Although microglia are necessary for normal function, uncontrolled and over-activated microglia can result in disastrous neurotoxic consequences. Indeed, microglia are a predominant source of pro-inflammatory mediators including cytokines, complement factors, free radicals, nitric oxide (NO), chemokines and prostanglandins, all of which potentially contribute to further neuronal dysfunction and death.^1, 2, 3^ Activation of microglia towards a pro-inflammatory phenotype and the resulting inflammatory response are typical features of neurodegenerative and neuroinflammatory disorders and have an important role in the demise of different neuronal populations. In fact, evidence from numerous clinical neuropathological observations and *in vivo* studies suggest a prominent role of activated microglia in the initiation and/or aggravation of neurodegenerative disorders, including Alzheimer's disease (AD) and Parkinson's disease (PD).^1, 3, 4, 5^

Caspases, a family of cysteinyl aspartate-specific proteases, are best known as executioners of apoptotic cell death and their activation are considered to be a commitment to cell death.^6^ However, certain caspases also function as regulatory molecules for immunity, cell differentiation and cell fate determination. We have characterized a novel and unexpected mechanism involved in the activation of microglia in response to different TLR4 ligands. This mechanism involves a caspase-dependent signaling governing microglia activation. We showed that the orderly activation of caspase-8 and caspase-3 (so-called apoptotic caspases) regulate microglia activation via a protein kinase C (PKC*δ*)-dependent pathway. We found that the stimulation of microglia with various TLR ligands activates caspase-8 and caspase-3 in microglia without triggering cell death *in vitro* and *in vivo*. Knockdown or chemical inhibition of each of these caspases hindered microglia activation. Moreover, we provided compelling evidence that these caspases are activated in microglia in the ventral mesencephalon of PD and the frontal cortex of AD subjects.^5^

One central question emerging from the discovery that the apoptotic caspases regulate microglia activation is, which mechanism prevents them from killing the microglia cells? In this study, we show that the control of caspase-3 cleavage by cellular inhibitor of apoptosis protein 2 (cIAP2) dictates whether microglia will undergo pro-inflammatory activation or apoptosis.

## Results

### Distinctive caspase-3 processing profile in microglia cells after pro-inflammatory stimulus

**Figure 1:**
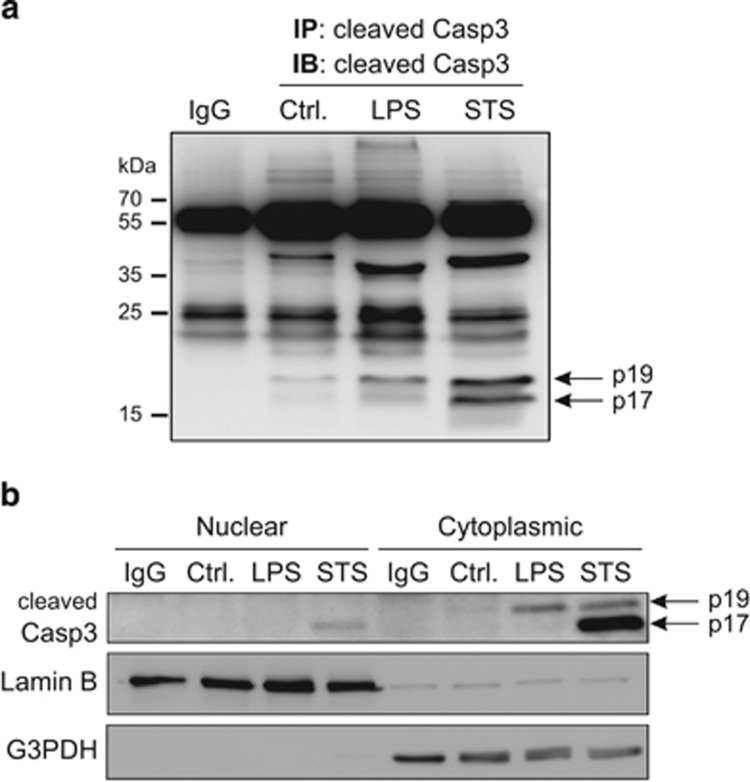
Distinctive caspase-3 processing profile in pro-inflammatory activated *versus* dying microglia. BV2 microglia cells were treated with 1 *μ*g/ml LPS for 24 h or 0.1 *μ*M STS for 3 h to promote pro-inflammatory activation or cell death, respectively. (**a**) Immunoblot analysis demonstrates presence of the p19 caspase-3 subunit in the immune complexes formed after pull down with cleaved caspase-3 Asp175 antibody, which recognizes both p17 and p19 subunits, in LPS-treated microglia. Immune complexes from STS-treated microglia contained both p17 and p19 caspase-3 subunits. (**b**) Subcellular fractionation illustrates the restricted cytoplasmic localization of p19 subunit, in contrast to the p17 subunit that was found to localize in both the cytoplasmic and the nuclear fractions

In an attempt to characterize the molecular events that hinder active caspase-3 from killing microglia, a biochemical analysis of caspase-3 processing was performed in activated microglia cells *versus* dying microglia cells. Caspase-3 is synthesized as a single-chain inactive zymogen, containing a prodomain, as well as large and small subunits that include the residues required for substrate recognition and cleavage. Caspase-3 activation occurs in two stages.^7^ First, caspase-3 proforms are cleaved by upstream caspases, such as active caspase-8, at Asp175 to generate intermediate, yet still active, heterotetramer complexes consisting of two p19 and two p12 peptides (p19/p12 complexes). The second stage involves removal of the short prodomain from the p19 peptides by autocatalytic processing, and cleavage at residue Asp28, to generate the fully mature p17/p12 form of the enzyme (see scheme in Figure 6). BV2 microglia cells were stimulated with lipopolysaccharide (LPS), the major component of Gram-negative bacterial walls and a ligand for TLR4, to investigate the processing of caspase-3 in activated microglia. Of note, intracerebral delivery of LPS, which leads *in vivo* to microglia activation and neuronal injury, is used as model for brain inflammation.^8, 9^ Immunoprecipitation using a polyclonal antibody raised against cleaved caspase-3 Asp175, which recognized both p17 and p19 subunit, was used to isolate and concentrate caspase-3 subunits. Subsequent immunoblot analysis using the same antibody revealed that upon LPS-induced microglia activation, the processing of the p19 N-terminal caspase-3 fragment containing the prodomain to the active p17 fragment is prevented (Figure 1a). In contrast to LPS treatment, exposure of BV2 microglia cells to a death stimulus such as staurosporine (STS) led to a significantly greater caspase-3 processing and appearance of the active p17 fragment (Figure 1a). This results are in agreement with the reported moderate D(OMe)E(OMe)VD(OMe)-ase (DEVD-ase) activity, which reflects caspase-3 function as protease, observed upon treatment of BV2 microglia cells with various pro-inflammogens, including LPS^5^ (Figure 3f).

### Cytoplasmic retention of active caspase-3 in pro-inflammatory stimulated microglia cells

We hypothesized that during microglia activation, the avoidance of p19 to p17 caspase-3 conversion controls both the strength of activity and the localization of caspase-3. It is worth noting that nuclear localization of active caspase-3 appears to be regulated by an active transport system.^10, 11^ In addition, removal of the short prodomain from caspase-3 fragments (i.e., conversion of p19 to p17) contributes to its nuclear relocalization.^12^ We previously reported using confocal cell imaging that following LPS treatment of microglia BV2 cells, cleaved caspase-3 Asp175 is found to be located outside the nuclear compartment.^5^ However, this experimental setup does not allow for the discrimination between the possible p17 and p19 caspase-3 subunits. To further characterize and reveal the subcellular localization of those caspase-3 subunits in activated microglia, immunoblot analysis for cleaved caspase-3 was performed on both nuclear and cytoplasmic subcellular fractions. The p19 subunit was predominantly found to be localized in the cytosol in both the activated and dying microglia. In contrast, the p17 subunit, which was detected in the dying STS-treated microglia, was detected in both the cytoplasmic and nuclear compartments (Figure 1b). Collectively, these data indicate the presence of cytoplasmic active caspase-3 in pro-inflammatory-activated microglia, in contrast to whole-cell (cytoplasm/nucleus) caspase-3 activation in the dying cells. These conclusions are further supported by our previous finding that PKC*δ*, a cytoplasmic caspase-3 substrate, but not poly(ADP-ribose) polymerase (PARP-1) a nuclear caspase-3 nuclear substrate, is found to be cleaved in microglia cells in response to LPS treatment.^5^

### Pro-inflammatory activation of microglia is associated with induction of caspase-3-interacting protein cIAP2

**Figure 2:**
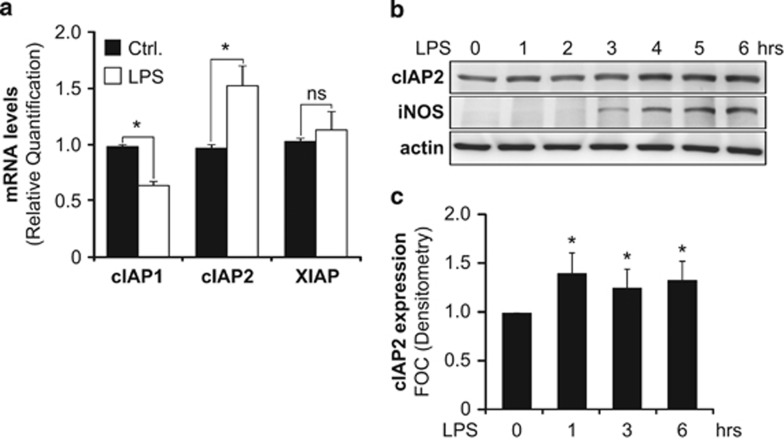
Levels of cIAP2 increase in BV2 microglia cells after LPS treatment. (**a**) mRNA levels of cIAP1, cIAP2 and XIAP were assessed by qPCR in untreated and 6 h LPS-treated BV2 microglia cells. (**b**) LPS-induced cIAP2 protein expression was analyzed by immunobloting for the indicated time points. iNOS expression was used as a marker for microglial pro-inflammatory activation and *β*-actin used as standard for equal loading of protein. (**c**) Quantification of cIAP2 protein expression is depicted for LPS-treated BV2 cells. Data are expressed as mean±S.E.M.; *n*=3; *P< 0.05

**Figure 3:**
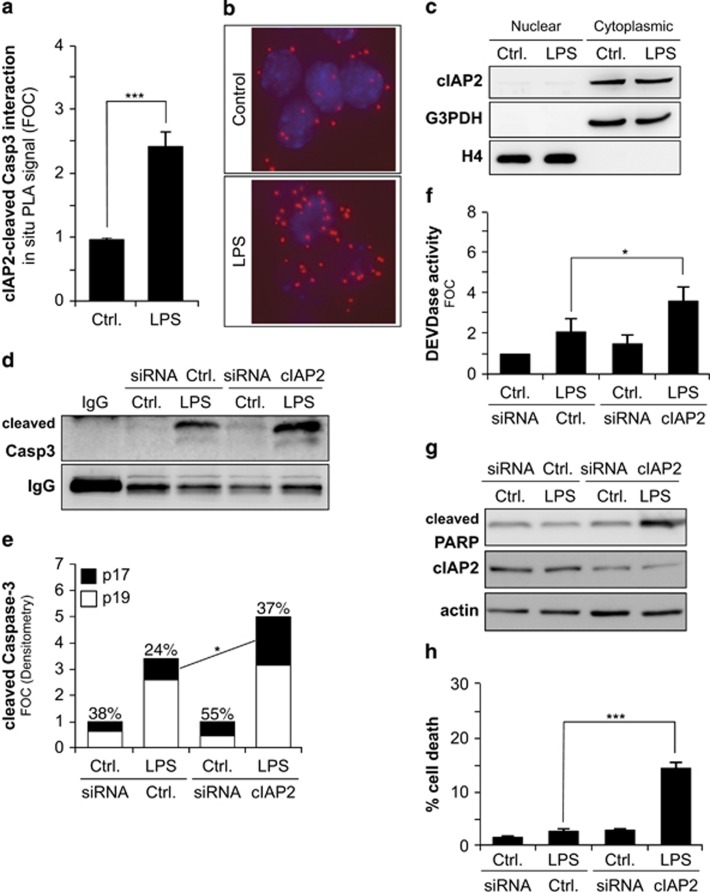
cIAP2 interaction with cleaved caspase-3 regulates the conversion of p19 into p17 caspase-3 subunit, its enzymatic activity and apoptosis in microglia. (**a**) Quantification of *in situ* PLA demonstrating protein interactions between cleaved caspase-3 Asp175 and cIAP2 occurring in LPS-treated BV2 microglia cells as compared with untreated cells. Data are presented as mean±S.E.M. of fluorescent dots/cell; *n*=3; ***P< 0.001. (**b**) Representative images of PLA in control and LPS-treated cells are depicted. Interactions between cIAP2 and cleaved caspase-3 Asp175 are shown as red dots. (**c**) Subcellular fractionation illustrates the cytoplasmic localization of cIAP2. (**d**) Immunoprecipitation and subsequent immunoblot using an anti-cleaved caspase-3 Asp175 antibody revealed that selective siRNAs knockdown of cIAP2 promotes caspase-3 p19 to p17 subunit conversion in LPS-treated BV2 cells. Non-targeting siRNAs were used as control. (**e**) Densitometry analysis of immunoblots showing the total levels of cleaved caspase-3 and the p17 to p19 ratio. Graphed values are mean of three independent experiments. Values above each bar represent the percentage of p17 fragment observed. Increased p19 to p17 conversion upon cIAP2 silencing was found to be statistically significant, *P<0.05 (**f**) DEVD-ase activity, (**g**) immunoblot against cleaved PARP, cIAP2 and *β*-actin and (**h**) cell death quantification in untreated or LPS-treated BV2 microglia cells after knockdown with non-targeting or cIAP2-targeting siRNAs pool. Data are expressed as mean±S.E.M.; *n*=3; *P*< 0.05, **P< 0.01

Thereafter, we aimed at elucidating the molecular mechanism that controls the restricted caspase-3 processing in microglia cells and thereby prevents their death and contributes to their activation towards a pro-inflammatory phenotype. Given the devastating consequences of caspase activation, cells have evolved mechanisms to regulate caspase activity. Inhibitors of apoptosis protein (IAPs) constitute an important class of apoptosis regulators functioning at the level of caspase activity.^13, 14^ They recognize, through baculovirus IAP repeat (BIR) domains, a specific sequence found on caspases called the IAP-binding motif (IBM). The IBM is found on the N-terminal of the p19 peptide but is absent (through autocatalytic cleavage) from the p17 peptide. X-linked IAP (XIAP), the prototypical IAP in mammals, inhibits apoptosis largely through direct inhibition of the initiator caspase-9 and the effector caspase-3 and caspase-7. Two additional IAP family members, cIAP1 and cIAP2, were once thought to also inhibit caspases, but more recent studies suggest that these IAPs bind to effector caspases but do not inhibit their proteolytic activities.^15, 16, 17^ It is very attractive to envisage that upregulation of IAPs during the caspase-mediated microglia activation is responsible for holding back caspase-3 in terms of activity and subcellular localization and therefore would be a key molecular event in preventing the cell death. Therefore, the mRNA expression levels of XIAP, cIAP1 and cIAP2 in microglia cells after LPS treatment was analyzed. Remarkably, only cIAP2 mRNA expression levels were found to be increased in microglia up to 6 h post LPS treatment (Figure 2a). Interestingly, cIAP1 mRNA expression levels were found to be reduced in LPS-treated microglia cells. Immunoblot analysis confirmed the increase in cIAP2 expression at protein levels in a time-dependent manner upon microglia activation in response to LPS treatment (Figures 2b and c). Worth a notice, LPS treatment has been reported to induce cIAP2 expression in macrophages.^18^ In addition, cIAP2-deficient mice exhibit profound resistance to LPS-induced sepsis.^19^ Caspase-3–cIAP2 interaction has previously been reported in various cellular model systems.^16, 20, 21^ Further, protein–protein interaction between caspase-3 and cIAP2 was confirmed in microglia using an *in situ* proximity ligation assay (PLA) (Figures 3a and b). The predominant cytoplasmic localization of cIAP2 was confirmed by immunoblot analysis performed on both the nuclear and cytoplasmic subcellular fractions (Figure 3c).

### cIAP2 controls the conversion of p19 into p17 caspase-3 subunit in microglia

As previously mentioned, the IBM is found in the short prodomain of caspase-3 and is present in the p19 peptide but is absent from the p17 peptide. Interestingly, the processing of the caspase-3 p19 fragment to the p17 fragment has been suggested to be controlled by cIAP2.^22^ To investigate the functional role of the caspase-3–cIAP2 association, we next determined the effect of cIAP2 silencing using small interfering RNA (siRNA) (Figures 3d and e) on the processing of caspase-3 upon LPS-induced BV2 microglia activation. Immunoprecipitation using the cleaved caspase-3 Asp175 antibody, which recognized p17 and p19 subunit, was used to isolate and concentrate caspase-3 subunits. Subsequent immunoblot analysis using the same antibody revealed that overall cleaved caspase-3 levels were increased and the generation of the caspase-3 p17 fragment in LPS-treated microglia was increased by 1.5-fold in cells transfected with a cIAP2 siRNA as compared with control siRNA (Figures 3d and e). DEVD-ase activity assay also revealed that cIAP2 siRNA positively affected caspase-3-like activity in LPS-treated microglia cells (Figure 3f).

### cIAP2 silencing promotes cell death and hinders microglia activation upon pro-inflammatory stimulus

**Figure 4:**
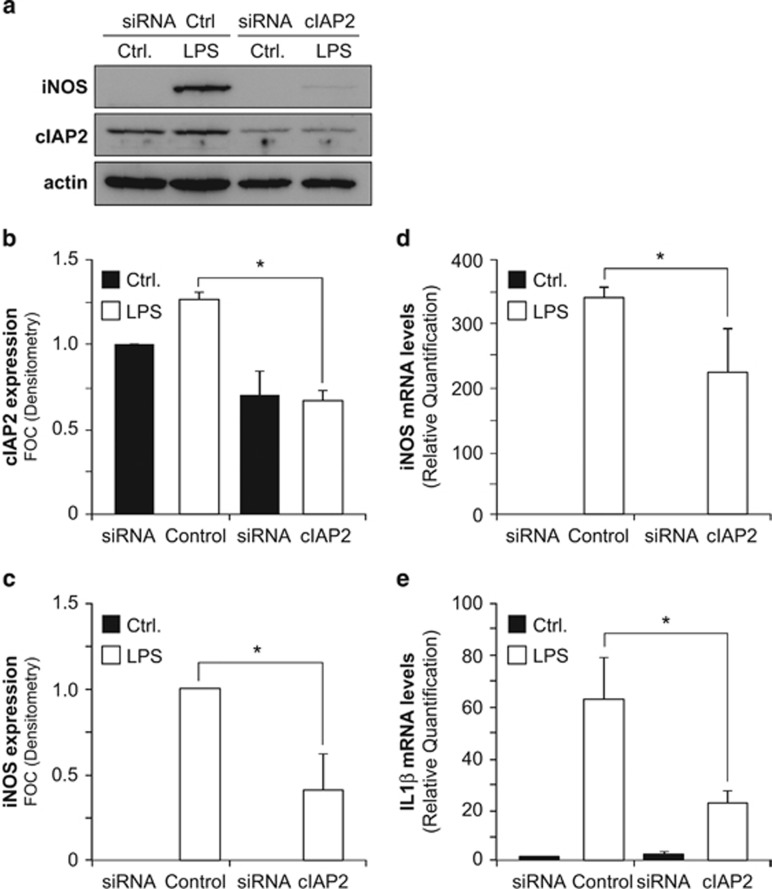
cIAP2 silencing hinders microglia pro-inflammatory activation upon LPS treatment. Knockdown of cIAP2 expression using siRNA prevents LPS-induced expression of iNOS in BV2 microglia cells. (**a**) Representative immunoblot using anti-iNOS, anti-cIAP2 and anti-actin antibodies. Quantification of (**b**) cIAP2 and (**c**) iNOS protein expression. (**d**) iNOS and (**e**) IL-1β mRNA expression levels were assessed by qPCR in untreated and LPS-treated BV2 microglia cells, transduced with non-targeting control or cIAP2 siRNAs. Values are average±S.E.M. of three independent experiments

The levels of cIAP2 can help to determine the threshold of caspase-3 activity that a cell can withstand before succumbing to the irreversible caspase cascade leading to apoptosis. Moreover, the control of caspase-3 p19 to p17 subunit conversion by cIAP2 can regulate the subcellular localization of active caspase-3, that is, p19/p12 complexes being cytoplasmic and p17/p12 complexes exhibiting the ability to relocate into the nucleus. The selective localization of active caspase-3 complexes can in turn regulate their ability to process cytoplasmic substrates (e.g., PKCδ) or nuclear substrates (e.g., PARP) and thereby affect different biological processes such as microglia pro-inflammatory activation or apoptotic cell death, respectively^5^ (see scheme in Figure 6). In agreement, increased PARP cleavage was observed in LPS-treated BV2 cells, where cIAP2 expression was targeted using an siRNA strategy (Figure 3g). As a result, increased cell death, as monitored by the appearance of fragmented, damaged or condensed nuclei and apoptotic phosphatidylserine (PS) exposure, was observed upon cIAP2 silencing in LPS-activated microglia (Figure 3h and supplementary Figure S1a). Following LPS treatment, BV2 microglia cells did show features of activated microglia, such as inducible NO synthase (iNOS) expression and production of cytokine interleukin-1*β* (IL-1*β*) (Figures 4a and e). Remarkably, when we transfected BV2 microglia cells with siRNA targeting cIAP2 (Figures 4a and b), LPS treatment failed to induce iNOS mRNA and protein expression (Figures 4a, c and d) and production of IL-1*β* cytokine (Figure 4e) as effectively. Collectively, these data indicated that cIAP2 silencing affects both the microglial cell death and activation under a pro-inflammatory stimulus like LPS. Thus, disruption of the cIAP2 control of microglial caspase-3 activation appears to exhibit pleotropic effects, possibly as a result of the promotion of p19 to p17 caspase-3 conversion, that should result in an increase of the p17/p12 complexes at the expense of the p19/p12 complexes, which regulate cell death and activation, respectively.

### SMAC mimetic BV6 enhances caspase-3 activation and promotes cell death of LPS-activated microglia

**Figure 5:**
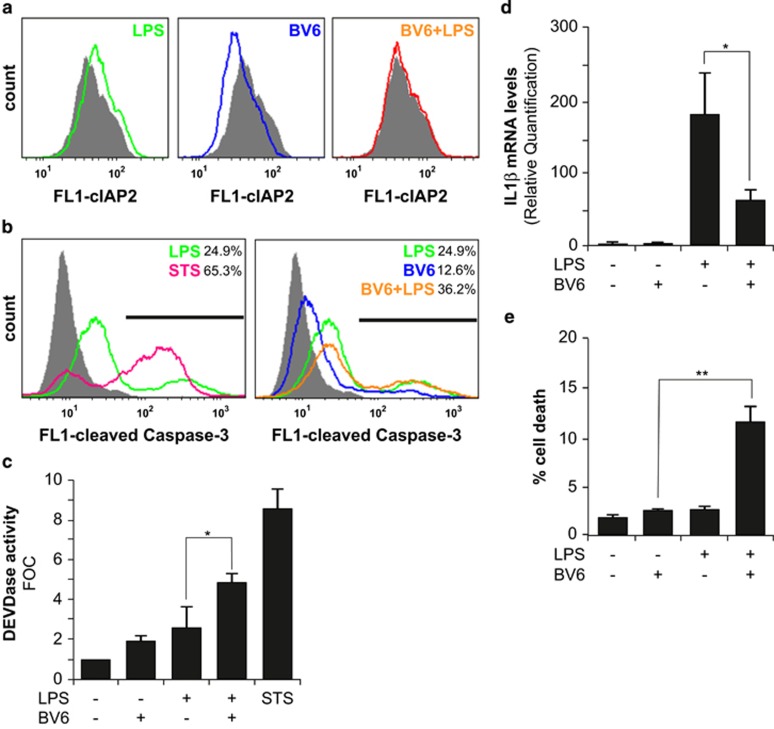
SMAC mimetic enhances caspase-3 activation and promotes cell death of LPS-activated microglia. BV2 microglia cells, pretreated or not with 1 *μ*M BV6 compound for 24 h, were subsequently treated with 1 *μ*g/ml LPS for 6 h. STS (0.1 *μ*M) for 3 h was used as cell death stimulus. (**a**) cIAP2 expression was assessed by FACS analysis. Pretreatment with the BV6 SMAC mimetic compound led to (**b**) increased appearance of cleaved caspase-3 Asp175 as seen by FACS analysis and (**c**) caspase-3 activity (DEVD-ase activity), (**d**) decreased IL-1*β* mRNA expression and (**e**) cell death as monitored by the appearance of fragmented, damaged or condensed nuclei in LPS-treated BV2 microglia cells. Data are expressed as mean±S.E.M.; *n*=3; *P< 0.05; **P<0.01

**Figure 6:**
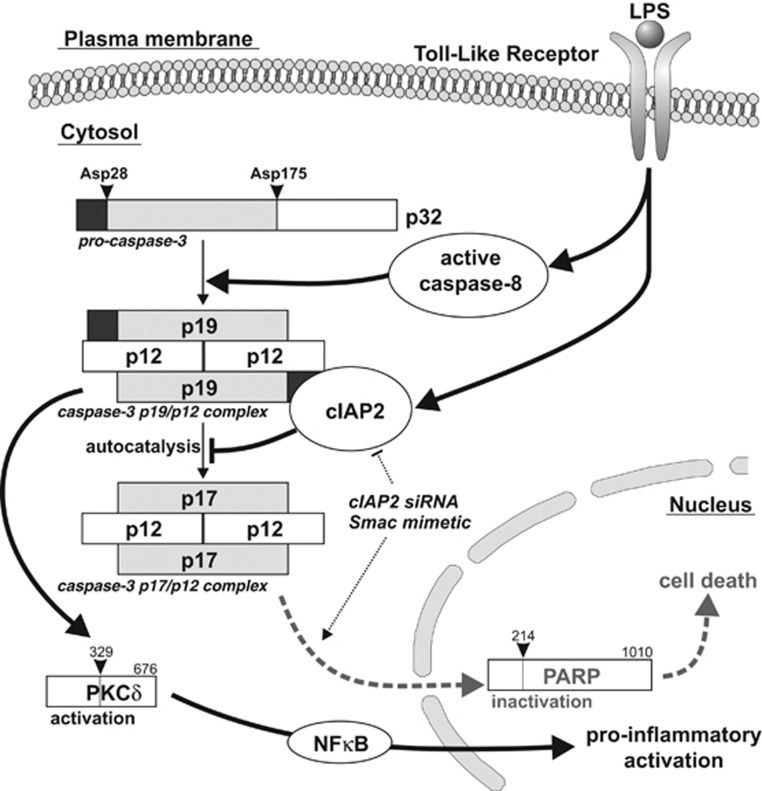
Scheme illustrating the effect of cIAP2 on the caspase-3 activation steps and consequently biological functions. Pro-caspase-3 is cleaved by upstream caspases, such as active caspases-8, at Asp175 to generate intermediate, yet still active, p19/p12 complexes. Thereafter, autocatalytic processing at residue Asp28 and removal of the short prodomain from the p19 peptides, generate p17/p12 complexes that form the fully mature form of the enzyme and translocate to the nucleus. In turn, p19/p12 and p17/p12 complexes can cleave substrates in the cytoplasmic or cytoplasmic/nuclear cell compartment, respectively, and thereby regulate pro-inflammatory activation or apoptotic cell death of the microglia cells. Upon pro-inflammatory stimulation, upregulated cIAP2 binds to caspase-3 prodomain and prevents the p19 to p17 caspase-3 subunit conversion

Mammalian cells possess natural antagonists of IAP-mediated caspase inhibition such as SMAC (second mitochondria-derived activator of caspases) also known as DIABLO (direct inhibitor of apoptosis-binding protein with low isoelectric point).^23, 24^ SMAC facilitates activation of apoptotic caspases by releasing these proteases from the inhibitory interaction with IAPs. In the case of XIAP, SMAC binding results in its dissociation from the bound caspases. In contrast, in the case of cIAP1/2, SMAC binding triggers autoubiquitination and proteasomal degradation of these molecules. Compounds mimicking the inhibitory effect of SMAC on IAPs, referred as SMAC mimetics, have been developed with the aim to achieve sensitization of cells to apoptotic cell death. BV6, a SMAC mimetic, has been shown to induce rapid proteasomal degradation of cIAPs, and thereby abrogate cIAPs-mediated inhibition of caspases, and induce cell death.^25, 26^ In view of the importance of cIAP2 in the regulation of caspase-3 processing upon pro-inflammatory activation of microglia, we decided to analyze the effects of the SMAC mimetic BV6 on BV2 microglia cells. We first selected a concentration of the BV6 compound (i.e., 1 *μ*M), which only induces moderate cell death in microglia, but reduces efficiently cIAP2 protein expression levels in those cells as investigated by fluorescence-activated cell sorting (FACS) analysis (Figure 5a). Furthermore, BV6 treatment was also efficient at counteracting LPS-induced cIAP2 expression in microglia. These results are in agreement with previous studies that demonstrated the proteolytic degradation of cIAP1/2 protein upon BV6 treatment in various cell types including myeloid cells.^27^ Noteworthy, the appearance of cleaved caspase-3 Asp175 and associated DEVD-ase activity were found increased in BV6-treated microglia cells. Moreover, BV6 treatment significantly enhanced these two parameters in LPS-treated microglia cells (Figures 5b and c). In contrast, decreased LPS-induced IL-1*β* expression was observed upon exposure to the BV6 compound (Figure 5d). Finally, in agreement with the above described negative impact of cIAP2 on caspase-3 processing towards the death-effective p17/p12 complexes, the SMAC mimetic BV6 promoted cell death of LPS-treated microglia (Figure 5e and supplementary Figure S1b and supplementary Figure S2). Altogether, these results demonstrated that the SMAC mimetic BV6 is indeed able to suppress the inhibitory effect of cIAP2 on full maturation of caspase-3 complexes and thereby promote microglial cell death (Figure 6).

## Discussion

There is compelling evidence that within the caspase family of cysteinyl aspartate-specific proteases, caspase-3 can perform as a key executor of the apoptotic cell death program.^28^ Activated caspase-3 leads, for example, to the processing of PARP, a caspase-3 nuclear substrate, and thereby contributes to demise of the cell. Nevertheless, increasing number of studies likewise call attention to the non-apoptotic functions for this so-called apoptotic caspase, including in the neuronal cell population. For instance, regulation of synaptic plasticity and function has been shown to be associated with caspase-3 activation.^29^ The non-apoptotic functions of caspase-3 in the brain are not limited to the neuronal population. Indeed, we recently uncovered an unexpected role for caspase-3 in the process of microglia pro-inflammatory activation.^5^ Activation of microglia and inflammation-mediated neurotoxicity are believed to have important roles in the pathogenesis of several neurodegenerative disorders. We showed that orderly activation of caspase-8 and caspase-3 regulates microglia activation through a PKC*δ*-dependent pathway. Thus, caspase-3 activation and subsequent proteolytic activation or inactivation of cytoplasmic (e.g., PKC*δ*) or nuclear (e.g., PARP) caspase-3 downstream targets can lead to rather contrasting effects, microglia pro-inflammatory activation or apoptotic cell death. What restricts caspase-3 activation in terms of subcellular localization and intensity might provide an answer to the conflicting functions of caspase-3 in microglia cells. We uncovered that different profiles of caspase-3 processing can be observed in activated *versus* dying microglia cells, that is, the p19 caspase-3 subunit seems to occur predominately in activated microglia, whereas the p17 caspase-3 subunit arises mostly during the apoptosis. Interestingly, the processing of the cytosolic caspase-3 p19 fragment to the p17 fragment, which has the ability to relocate into the nuclear compartment, has been suggested to be controlled by cIAP2.^22^ We and others report that cIAP2 protein expression is upregulated in microglia and macrophages upon treatment with a proinflammogen such as LPS.^19^ In addition, cIAP2-deficient mice have been reported to exhibit profound resistance to LPS-induced sepsis, specifically because of an attenuated inflammatory response.^19^ We demonstrate that counteracting the repressive effect of cIAP2 on caspase-3 activation, using siRNA targeting cIAP2 itself or a SMAC mimetic such as the BV6 compound, reduced the pro-inflammatory activation of microglia cells and promoted their death. On the basis of our findings, we propose that the different caspase-3 functions in microglia, and potentially other cell types, resides in the active caspase-3 complexes formed (Figure 6). Cytoplasmic caspase-3 p19/p12 complexes would be responsible for the pro-inflammatory activation of microglia, whereas the nuclear caspase-3 p17/p12 complexes would promote apoptosis in microglia. Collectively, our data reveal that cIAP2 may work as a molecular switch for the decision between pro-inflammatory activation and apoptosis in microglia. These results also could indicate cIAP2 as a possible therapeutic target to modulate microglia pro-inflammatory activation and associated neurotoxicity observed, for example, in neurodegenerative disorders. SMAC mimetics, which oppose the effect of cIAP2, would also be seen as potential candidate drugs. In fact, SMAC mimetics, alone or in association, are already considered as a new class of targeted drugs for the treatment of solid tumors and hematologic cancers with ongoing clinical trials (www.clinicaltrials.gov).^30^ Our results further illustrate the potential impact of SMAC mimetics on microglia-associated brain diseases, comporting a neuroinflammatory component, such as neurodegenerative disorders, brain trauma or even stroke. However, the benefit of killing microglia cells, instead of suffering their pro-inflammatory activation remains to be proven and would require further investigations. In summary, we show that the cIAP2 is involved in regulating caspase-3 activation and thereby functions in microglia. We conclude that regulation of the conversion of caspase-3 p19 subunit to p17 subunit in those cells can also be used to modulate microglial biological functions.

## Materials and Methods

### Cell culture, transfection and treatments

Murine microglial BV2 cell line was cultured as described.^31^ Cells were maintained in DMEM medium (Gibco BRL, Carlsbad, CA, USA) supplemented with penicillin (100 *μ*g/ml), streptomycin (100 *μ*g/ml) and 10% fetal calf serum (FCS), reduced to 5% FCS while performing the experiments. Transfection of BV2 cells was carried out using Lipofectamine 2000 (Invitrogen, Carlsbad, CA, USA) following the manufacturer's recommendation. Predesigned and tested ON-TARGETplus SMARTpool siRNA against mouse BIRC3 (NM-007464; #L-062425-00-0005) and non-targeted sequence (#D-001810-01-05) were purchased from Thermo Scientific (Walthman, MA, USA). Cells were treated with 1 *μ*g/ml LPS (from *Escherichia coli*, serotype 026:B6; Sigma-Aldrich, St. Louis, MO, USA) or 0.1 *μ*M STS (Sigma-Aldrich) for the indicated period of time. BV6, pan-IAP antagonist bivalent compound (used at 1 *μ*M), was generously provided by Genentech, Inc. (South San Francisco, CA, USA).^25^

### Protein extracts, immunoprecipitation and immunoblotting

Cytoplasmic and nuclear protein fractions were extracted in subcellular fractionation buffer (150 mM NaCl, 50 mM HEPES pH 7.4, 0.5% NP40, 2 mM PMSF and Protease Inhibitor Cockail) alternatively as reported by Joseph *et al.*^32^ Total protein extracts were made directly in Laemmli buffer. For immunoprecipitation, cells were lysed in an IP lysis buffer (20 mM Tris-HCl pH 7.5, 140 mM NaCl, 1% Triton-X100, 2 mM EDTA, 1 mM PMSF, 10% glycerol and Protease Inhibitor Cocktail) for 15 min before sonication. Protein G Sepharose (GE Healthcare, Uppsala, Sweden) precleared total protein extracts were incubated with the cleaved caspase-3 (Asp175) rabbit polyclonal antibody in IP lysis buffer overnight at 4 °C. Normal rabbit IgG (# AB-105-C, R&D Systems, Minneapolis, MN, USA) was used as control. Immunocomplexes bound to protein G Sepharose were collected by centrifugation and washed in IP wash buffer (50 mM Tris-HCl, pH 7.5, 0.1% SDS, 1% NP40 and 62.5 mM NaCl). For immunoblot analysis, protein extracts were resolved on 12 or 15% SDS-polyacrylamide gel electrophoresis and then blotted onto nitrocellulose membrane. Membranes were blocked in 5% milk and incubated with antibodies raised against cIAP2 (Santa Cruz Biotechnology, Santa Cruz, CA, USA), cleaved caspase-3 (Asp175) (Cell Signaling, Beverly, MA, USA), cleaved PARP (Cell Signaling), G3PDH (Trevigen, Gaithersburg, MD, USA), iNOS (Santa Cruz Biotechnology), Lamin B (Abcam, Cambridge, MA, USA) or H4 (Active Motif, Carlsbad, CA, USA), overnight at 4 °C, followed by incubation with the appropriate horseradish peroxidase secondary antibody (Pierce, Rockford, IL, USA, 1 : 10 000) for 1 h at room temperature. Immunoblot with anti-*β*-actin antibody (Sigma) was used for standardization of protein loading. Bands were visualized by enhanced chemiluminescence (ECL-Plus, Pierce) following the manufacturer's protocol. Densitometry was done using ImageJ software (NIH, USA; http.//imagej.nih.gov/ij).

### RNA isolation, cDNA synthesis and qPCR

RNA was isolated from 2 × 10^5^ cells using the total RNA extraction kit (Qiagen, Courtaboeuf, France). cDNA was synthesized from 1 *μ*g RNA using Oligo dT, dNTPs and Superscript II (Invitrogen). qPCR was performed using Sybr Green reagents (Applied Biosystems, Waltham, MA, USA) and the following primers (forward; reverse): NOS2 (5ʹ-GTGGTGACAAGCACATTTGG-3ʹ; 5ʹ-AAGGCCAAACACAGCATACC-3ʹ), IL-1*β* (5ʹ-GCTGCTTCCAAACCTTTGAC-3ʹ; 5ʹ-TTCTCCACAGCCACAATGAG-3ʹ), cIAP1/BIRC2 (5ʹ-TGATGGTGGCTTGAGATGTTGGGA-3ʹ; 5ʹ-TGAAT CTCATCAACAAACTCCTGACCC-3ʹ), cIAP2/BIRC3 (5ʹ-TGTCAGCCAAGTTCAAGCTG-3ʹ; 5ʹ-ATCTTCCGAACTTTCTCCAGGG-3ʹ), XIAP (5ʹ-CCATGTGTAGTGAAGAAGCCAGAT-3ʹ; 5ʹ-GATCATCAGCCCCTGTGTAGTAG-3ʹ) and *β*-actin (5ʹ-TTGCTGACAGGATGCAGAAG-3ʹ; 5ʹ-TGATCCACATCTGCTGGAAG-3ʹ). Results were calculated using delta Ct method.

### Caspase activity assay

Changes in caspase activity in microglia were measured using a luciferase-based assay from Promega (Stockholm, Sweden) known as Caspase-Glo (G890 for caspase-3/7). Equal volumes of cells and kit component were mixed onto a 96-well plate and incubated for 1 h at room temperature. The plate was analyzed using a luminometer and the value obtained normalized with the number of cells at harvest.

### Cell death quantification

After treatment, cells were collected and cytospins were prepared. Subsequently, DNA was stained with Hoechst 33342 (2 *μ*g/ml; Molecular Probes/Invitrogen, Carlsbad, CA, USA). The number of dying cells was measured quantitatively by assessing the percentage of cells with fragmented, damaged or condensed nuclei.

### FACS analysis

Appearance of subdiploid-fragmented nuclei derived from apoptotic cells were analyzed after DNA staining with 0.5 *μ*g/ml propidium iodide, in the presence of 2.5 *μ*g/ml RNase A in PBS, pH 7.4. Cleaved caspase-3 or cIAP2 expression was quantified in 0.25% paraformaldehyde-fixed cells following described protocol.^5^ Alexa Fluor 488-conjugated secondary antibodies (Molecular Probes/Invitrogen) were used for detection in the FL1 channel. For determination of the external exposition of PS residues, staining with Annexin-V-FITC and propidium iodide was used according to the manufacturer's instructions (#V13242, FITC Annexin V/Dead Cell Apoptosis kit, Invitrogen). Data are depicted in logarithmic scales. Analyses were carried out on a FACScalibur flow cytometer equipped with Cell Quest software (Becton Dickinson, Franklin Lakes, NJ, USA).

### *In situ* PLA

Cells were seeded on coverslips and treated as indicated. After treatment, cells were fixed with 4% paraformaldehyde in PBS. Protein–protein interactions between cIAP2 and cleaved caspase-3 in fixed cells were detected using the Duolink II *in situ* PLA from Olink Bioscience (Uppsala, Sweden). PLA was performed in a humidity chamber. After incubation with the blocking buffer (3% BSA and 0.3% Triton-X in PBS), cells were incubated with the primary antibodies mouse anti-cIAP2 (1 : 5000, R&D Systems) and rabbit anti-cleaved caspase-3 (1 : 400, Cell Signaling) antibodies, in the antibody diluent medium overnight at 4 °C. Cells were washed with supplied buffer A and incubated for 1 h in a humidity chamber at 37 °C with PLA probes detecting mouse or rabbit antibodies (Duolink II PLA probe anti-rabbit plus and Duolink II PLA probe anti-mouse minus diluted in the antibody diluent to a concentration of 1 : 5). After washing with buffer A, cells were incubated for 30 min at 37 °C with the ligation solution (Duolink II Ligation stock 1 : 5 and Duolink II Ligase 1 : 40). If the two protein targets are in close proximity, a template is formed for amplification. Detection of the amplified probe was done with the Duolink II Detection Reagents Red Kit. After repeated washing at room temperature with wash buffer B, coverslips were mounted onto slides using mounting medium containing DAPI and samples were observed using an epifluorescence microscope. Protein–protein interaction was measured as the number of fluorescent dots/cell analyzed with Duolink Image tool.

### Statistical analysis

Statistical analysis was performed using Student's *t*-test. *P*<0.05 was considered as statistically significant.

## Abbreviations

AD: Alzheimer's disease
BIR: baculovirus IAP repeat
cIAP: cellular inhibitor of apoptosis protein
IAP: inhibitor of apoptosis protein
IBM: IAP-binding motif
LPS: lipopolysaccharide
NO: nitric oxide
PARP: poly(ADP-ribose) polymerase
PD: Parkinson's disease
PKC: protein kinase C
siRNA: small interfering RNA
SMAC: second mitochondria-derived activator of caspases
STS: staurosporine
TLR: toll-like receptor
XIAP: X-linked IAP
