# Supplementary material for: Regulation of caspase-3 processing by cIAP2 controls the switch between pro-inflammatory activation and cell death in microglia
Source: Cell Death Dis. 2014 Dec 11;5(12):e1565–. doi: 10.1038/cddis.2014.514 (PMC4454160; doi:10.1038/cddis.2014.514)
Supplement: Supplementary Figures [file cddis2014514x1.doc]

**Supplementary Figure S1 | cIAP2 silencing or SMAC mimetic promotes PS exposure in LPS-activated microglia.** (**a**) Selective siRNAs knockdown of cIAP2 promotes PS exposure in 6 h LPS-treated BV2 microglia cells, as monitored by FACS analysis. Non-targeting siRNAs were used as control. (**b**) BV2 cells pretreated or not with 1µM BV6 compound for 24 h, were subsequently treated with 1µg/ml LPS for 6 h and PS exposure analyzed. Representative experiments of three independent biological replicates are depicted.

**Supplementary Figure S2 | SMAC mimetic promotes appearance of a subG1 peak in LPS-activated microglia.** Pretreatment with the BV6 SMAC mimetic compound led to cell death as monitored by the appearance of a subG1 peak by FACS analysis, in LPS-treated BV2 microglia cells. Data are expressed as mean ± s.e.m. (*n* = 3); **P* < 0.05.
